# Learning and exploration in action-perception loops

**DOI:** 10.3389/fncir.2013.00037

**Published:** 2013-03-22

**Authors:** Daniel Y. Little, Friedrich T. Sommer

**Affiliations:** ^1^Department of Molecular and Cell Biology, Redwood Center for Theoretical Neuroscience, University of CaliforniaBerkeley, CA, USA; ^2^Redwood Center for Theoretical Neuroscience, Helen Wills Neuroscience Institute, University of CaliforniaBerkeley, CA, USA

**Keywords:** knowledge acquisition, information theory, control theory, machine learning, behavioral psychology, computational neuroscience

## Abstract

Discovering the structure underlying observed data is a recurring problem in machine learning with important applications in neuroscience. It is also a primary function of the brain. When data can be actively collected in the context of a closed action-perception loop, behavior becomes a critical determinant of learning efficiency. Psychologists studying exploration and curiosity in humans and animals have long argued that learning itself is a *primary* motivator of behavior. However, the theoretical basis of learning-driven behavior is not well understood. Previous computational studies of behavior have largely focused on the control problem of maximizing acquisition of rewards and have treated learning the structure of data as a *secondary* objective. Here, we study exploration in the absence of external reward feedback. Instead, we take the quality of an agent's learned internal model to be the primary objective. In a simple probabilistic framework, we derive a Bayesian estimate for the amount of information about the environment an agent can expect to receive by taking an action, a measure we term the predicted information gain (PIG). We develop exploration strategies that approximately maximize PIG. One strategy based on value-iteration consistently learns faster than previously developed reward-free exploration strategies across a diverse range of environments. Psychologists believe the evolutionary advantage of learning-driven exploration lies in the generalized utility of an accurate internal model. Consistent with this hypothesis, we demonstrate that agents which learn more efficiently during exploration are later better able to accomplish a range of goal-directed tasks. We will conclude by discussing how our work elucidates the explorative behaviors of animals and humans, its relationship to other computational models of behavior, and its potential application to experimental design, such as in closed-loop neurophysiology studies.

## 1. Introduction

Computational models of exploratory behavior have largely focused on the role of exploration in the acquisition of external rewards (Thrun, 1992; Kaelbling et al., 1996; Sutton and Barto, 1998; Kawato and Samejima, 2007). In contrast, a consensus has emerged in behavioral psychology that learning represents the primary drive underlying explorative behaviors (Archer and Birke, 1983; Loewenstein, 1994; Silvia, 2005; Pisula, 2009). The computational principles underlying learning-driven exploration, however, have received much less attention. To address this gap, we introduce here a mathematical framework for studying how behavior affects learning and develop a novel model of learning-driven exploration.

Machine learning techniques for extracting the structure underlying sensory signals have often focused on passive learning systems that can not directly affect the sensory input. Exploration, in contrast, requires actively pursuing useful information and can only occur in the context of a closed action-perception loop. Learning in closed action-perception loops differs from passive learning both in terms of “what” is being learned as well as “how” it is learned (Gordon et al., 2011). In particular, in closed action-perception loops:

1. Sensorimotor contingencies must be learned.
2. Actions must be coordinated to direct the acquisition of data.

Sensorimotor contingencies refer to the causal role actions play on the sensory inputs we receive, such as the way visual inputs change as we shift our gaze or move our head. They must be taken into account to properly attribute changes in sensory signals to their causes. This tight interaction between actions and sensation is reflected in the neuroanatomy where sensory-motor integration has been reported at all levels of the brain (Guillery, 2005; Guillery and Sherman, 2011). We often take our implicit understanding of sensorimotor contingencies for granted, but in fact they must be learned during the course of development (the exception being contingencies for which we are hard-wired by evolution). This is eloquently expressed in the explorative behaviors of young infants (e.g., grasping and manipulating objects during proprioceptive exploration and then bringing them into visual view during intermodal exploration) (Rochat, 1989; O'Regan and Noë, 2001; Noë, 2004).

Not only are actions part of “what” we learn during exploration, they are also part of “how” we learn. To discover what is inside an unfamiliar box, a curious child must open it. To learn about the world, scientists perform experiments. Directing the acquisition of data is particularly important for embodied agents whose actuators and sensors are physically confined. Since the most informative data may not always be accessible to a physical sensor, embodiment may constrain an exploring agent and require that it coordinates its actions to retrieve useful data.

In the model we propose here, an agent moving between discrete states in a world has to learn how its actions influence its state transitions. The underlying transition dynamics is governed by a Controllable Markov Chain (CMC). Within this simple framework, various utility functions for guiding exploratory behaviors will be studied, as well as several methods for coordinating actions over time. The different exploratory strategies are compared in their rate of learning and how well they enable agents to perform goal-directed tasks.

## 2. Methods

### 2.1. Mathematical framework for embodied active learning

*CMCs* are a simple extension of Markov chains that incorporate a control variable for switching between different transition distributions in each state, e.g., (Gimbert, 2007). Formally, a CMC is a 3-tuple (
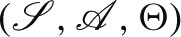
) where:

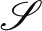
 is a finite set of *states* (here representing, the possible locations of an agent in its world) 
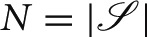
.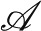 is a finite set of control values, or *actions*, an agent can choose from 
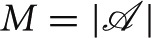
.
- Θ is a 3-dimensional *CMC kernel* describing the transition probabilities between states for each action (for example, the probability an agent moves from an *originating state s* to a *resultant state s*′ when it chooses action *a*):
  (1) $$\begin{array}{l} p(s^{\prime }|a,s;\Theta )=\Theta_{ass^{\prime }}\\ \;\Theta_{as\cdot }\in \Delta_{N\;-\;1} \end{array}$$

Here, Δ_*N* − 1_ denotes the standard (*N* − 1)-simplex and is used to constrain Θ to describing legitimate probability distributions:

$$\Delta_{N\;-\;1}:=\{(x_{0},x_{1},\ldots ,x_{N\;-\;1})\in {\mathbb{R}}^{N}|\sum_{i\;=\;0}^{N\;-\;1}x_{i}=1\;and\;x_{i}\geq 0\;\forall i\}$$

CMCs provides a simple mathematical framework for modeling exploration in embodied action-perception loops. At each time step, an exploring agent is allowed to select any action 
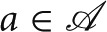
. This action, along with the agent's current state, then determines which transition distribution its next state is drawn from. For this study, we will make the simplifying assumption that the states can be directly observed by the agent, i.e., the system is not hidden. Since we are interested in the role behavior plays in learning about the world, we consider the exploration task of the agent to be the formation of an accurate estimate, or *internal model* $\hat{\Theta }$, of the true CMC kernel that describes its *world* Θ.

This framework captures the two important roles actions play in embodied learning. First, transitions depend on actions, and actions are thus a constituent part of “what” is being learned. Second, an agent's immediate ability to interact with and observe the world is limited by its current state. This restriction models the *embodiment* of the agent, and actions are “how” an agent can overcome this constraint on accessing information. Our primary question will be how action policies can optimize the speed and efficiency of learning in embodied action-perception loops as modeled by CMCs.

### 2.2. Information-theoretic assessment of learning

Following Pfaffelhuber (1972), we define *missing information* I_M_ as a measure of the inaccuracy of an agent's internal model. To compute I_M_, we first calculate the Kullback–Leibler (KL) divergence of the internal model from the world for each transition distribution:

(2) $${\text{D}}_{\text{KL}}(\Theta_{as\cdot }∥{\hat{\Theta }}_{as\cdot }):=\sum_{s^{\prime }=1}^{N}\Theta_{ass^{\prime }}\log_{2}(\frac{\Theta_{ass^{\prime }}}{{\hat{\Theta }}_{ass^{\prime }}})$$

The KL-divergence is an information-theoretic measure of the difference between two distributions. Specifically, Equation (2) gives the expected number of bits that would be lost if observations (following the true distribution) were communicated using an encoding scheme optimized for the estimated distribution (Cover and Thomas, 1991). It is large when the two distributions differ greatly and zero when they are identical. Next, missing information is defined as the unweighted sum of the KL-divergences: 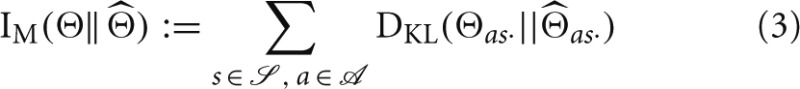


We will use missing information to assess learning under different explorative strategies. Steeper decreases in missing information over time represent faster learning and thus more efficient exploration. The definition of missing information and those of several other relevant terms that will be introduced later in this manuscript have been compiled into Table 1 for easy reference.

**Table 1:** **Table of measures**.

**Name used here, abbreviation (Equation)**	**Name used in (References)**	**Mathematical expression**
Missing information, I_M_ (3)	Missing information (Pfaffelhuber, 1972)	$\sum_{s,\;a}{\text{D}}_{\text{KL}}\left(\Theta_{as\cdot }||{\hat{\Theta }}_{as\cdot }\right)$
Information gain, I_G_ (6)		${\text{I}}_{\text{M}}(\Theta ∥\hat{\Theta })-{\text{I}}_{\text{M}}(\Theta ∥{\hat{\Theta }}^{a,\;s\to s*})$
Predicted information gain, PIG (7)	Information gain (Nelson, 2005)	$\sum_{s^{*}}{\hat{\Theta }}_{ass^{*}}{\text{D}}_{\text{KL}}({\hat{\Theta }}_{as\cdot }^{a,\;s\to s^{*}}∥{\hat{\Theta }}_{as\cdot })$
Posterior expected information gain, PEIG (17)	KL-divergence (Storck et al., 1995)	${\text{D}}_{\text{KL}}({\hat{\Theta }}_{as\cdot }^{\text{current}}∥{\hat{\Theta }}_{as\cdot }^{\text{past}})$
Predicted mode change, PMC (11)	Probability gain (Nelson, 2005)	$\sum_{s^{*}}{\hat{\Theta }}_{ass^{*}}\left[\max_{s^{\prime }}{\hat{\Theta }}_{ass^{\prime }}^{a,\;s\to s^{*}}-\max_{s^{\prime }}{\hat{\Theta }}_{ass^{\prime }}\right]$
Predicted *L*_1_ change, PLC (12)	Impact (Nelson, 2005)	$\sum_{s^{*}}{\hat{\Theta }}_{ass^{*}}\left[\frac{1}{N}\sum_{s^{\prime }}\left|{\hat{\Theta }}_{ass^{\prime }}^{a,\;s\to s^{*}}-{\hat{\Theta }}_{ass^{\prime }}\right|\right]$

### 2.3. Bayesian inference learning

As an agent acts in its world, it observes the state transitions and can use these observations to update its internal model $\hat{\Theta }$. Taking a Bayesian approach, we assume the agent models its world Θ as a random variable **Θ** with an initial *prior distribution f* over the space of possible CMC structures, 
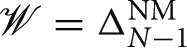
. There is no standard nomenclature for tensor random variables and we will therefore use a bold upright theta **Θ** to denote the random variable and a regular upright theta Θ to denote an arbitrary realization of this random variable. Thus, *f*(Θ) describes the exploring agent's initial belief that Θ accurately describes its world, i.e., that **Θ** = Θ. By Bayes' theorem, an agent can calculate a posterior belief on the structure of its world from its prior and any data it has collected, $\vec{\text{d}}$:

(4) $$f(\Theta |\vec{\text{d}})=\frac{p(\vec{\text{d}}|\Theta )f(\Theta )}{p(\vec{\text{d}})}$$

Bayes' theorem decomposes the posterior distribution of the CMC kernel into the likelihood function of the data, $p(\vec{\text{d}}|\Theta )$, and the prior, *f*(Θ). The normalization factor is calculated by integrating the numerator over 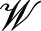: 
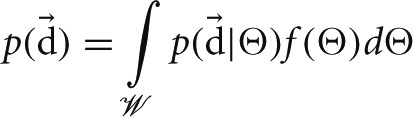


We now formulate a Bayesian estimate by directly calculating the posterior belief in transitioning to state *s*′ from state *s* under action *a*: 
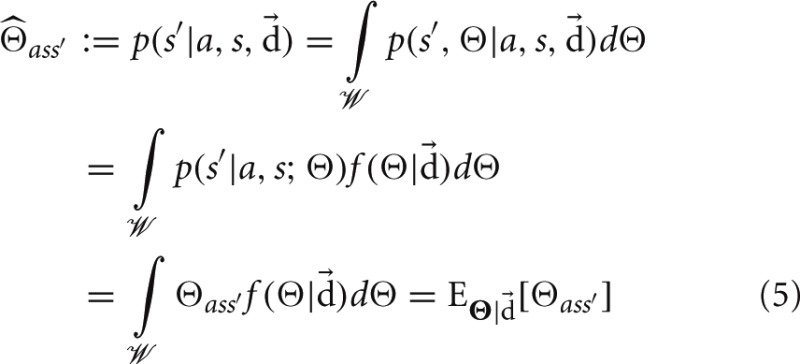


For discrete priors the above integrals would be replaced with summations. Equation (5) demonstrates that the Bayesian estimate is simply the expectation of the random variable given the data. While other estimates are possible for inferring world structure, such as Maximum Likelihood, the Bayesian estimate is often employed to avoid over-fitting (Manning et al., 2008). Moreover, as the following theorem demonstrates, the Bayesian estimate is optimal under our minimum missing information objective function.

**Theorem 1.** *Consider a CMC random variable* **Θ** *modeling the ground truth environment Θ and drawn from a prior distribution f. Given a history of observations* $\vec{\text{d}}$*, the expected missing information between* **Θ** *and an agent's internal model Φ is minimized by the Bayesian estimate Φ =* $\hat{\Theta }$*. That is:*

$$\hat{\Theta }:={\text{E}}_{\Theta \text{|}\vec{\text{d}}}[\Theta ]=\underset{\Theta }{\arg\;\min}{\text{E}}_{\Theta \text{|}\vec{\text{d}}}[{\text{I}}_{\text{M}}(\Theta ∥\Phi )]$$

*Proof.* See Appendix A1

The exact analytical form for the Bayesian estimate will depend on the prior distribution. We emphasize that the utility of the Bayesian estimate rests on the accuracy of its prior. In the discussion, we will address issues deriving from uncertain or inaccurate prior beliefs, but for now will provide the agents with priors that match the generative process by which we create new worlds for the agents to explore.

### 2.4. Three test environments for studying exploration

**Figure 1:**
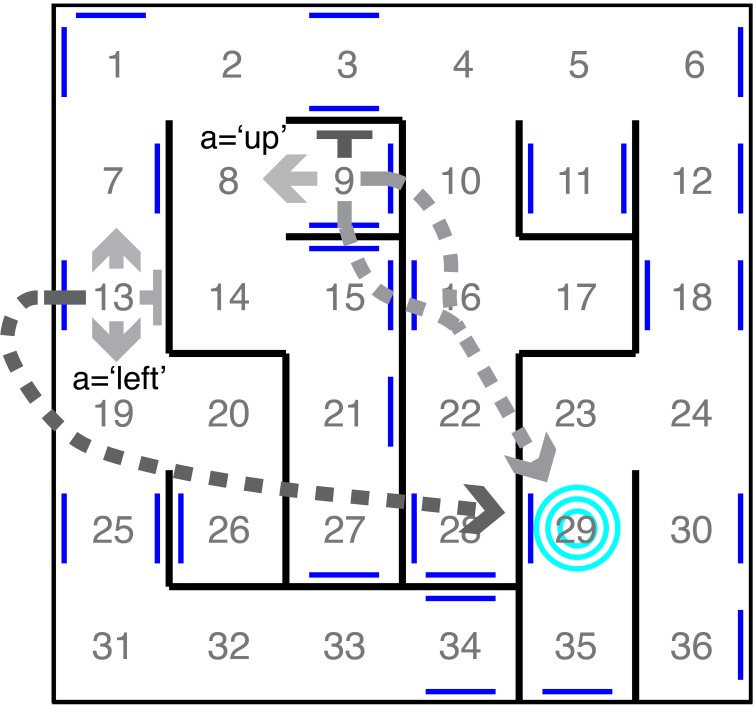
**Example maze.** The 36 states correspond to rooms in a maze. The four actions correspond to noisy translations in the cardinal directions. Two transition distributions are depicted, each by a set of four arrows emanating from their originating states. Flat-headed arrows represent translations into walls, resulting in staying in the same room. Dashed arrows represent translation into a portal (blue lines) leading to the absorbing state (blue target). The shading of an arrow indicates the probability of the transition (darker color represents higher probability).

**Figure A1:**
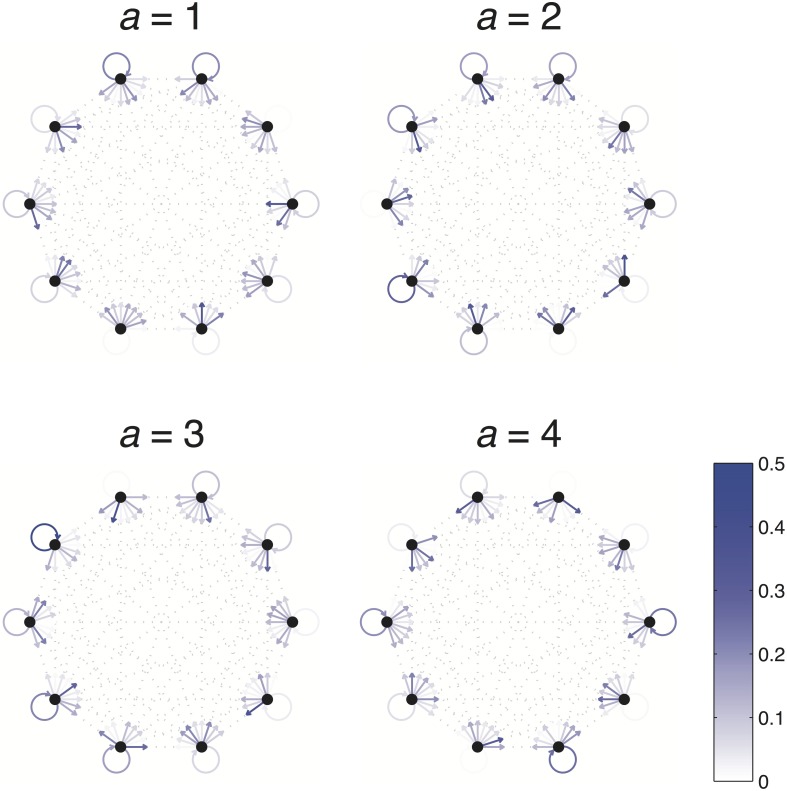
**Example Dense World.** Dense Worlds consist of 4 actions (separately depicted) and 10 states (depicted as nodes of the graphs). The transition probabilities associated with taking a particular action are depicted as arrows pointing from the current state to each of the possible resultant states. Arrow color depicts the likelihood of each transition.

**Figure A2:**
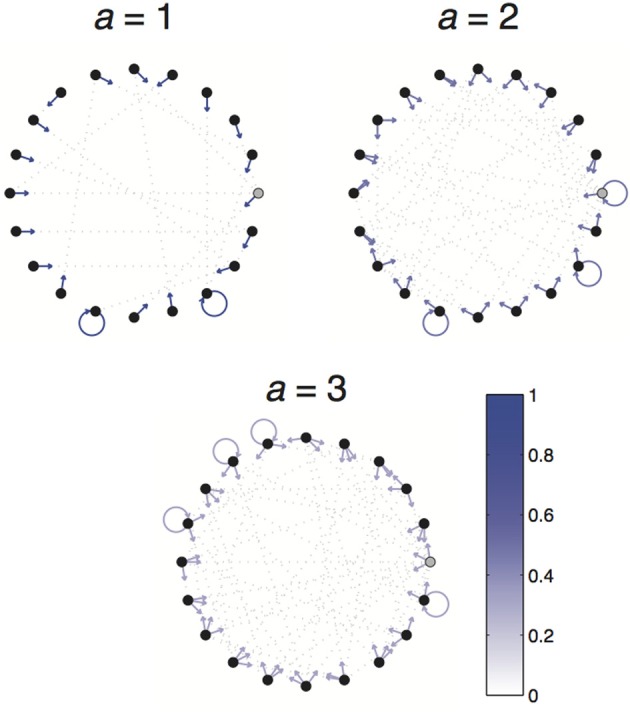
**Example 1-2-3 World.** 1-2-3 Worlds consist of 3 actions (separately depicted) and 20 states (depicted as nodes of the graphs). The transition probabilities associated with taking a particular action are depicted as arrows pointing from the current state to each of the possible resultant states. Arrow color depicts the likelihood of each transition. The absorbing state is depicted in gray.

In the course of exploration, the data an agent accumulates will depend on both its behavioral strategy as well as the structure of its world. We reasoned that studying diverse environments, i.e., CMCs that differ greatly in structure, would allow us to investigate how world structure effects the relative performance of different exploratory strategies and to identify action policies that produce efficient learning under broad conditions. We thus constructed and considered three classes of CMCs that differ greatly in structure: Dense Worlds, Mazes, and 1-2-3 Worlds. Dense Worlds are randomly generated from a uniform distribution over all CMCs with *N* = 10 states and *M* = 4 actions (see Figure A1 in Appendix). They therefore represent very unstructured worlds. Mazes, in contrast, are highly structured and model moving between rooms of a 6-by-6 maze (see Figure 1). The state space in mazes consist of the *N* = 36 rooms. The *M* = 4 actions correspond to the noisy translations in the four cardinal directions. To make the task of learning in mazes harder, 30 transporters are randomly distributed amongst the walls which lead to a randomly chosen absorbing state (concentric rings in Figure 1). While perhaps not typically abundant in mazes, absorbing states, such as at the bottom of a gravity well, are common in real world dynamics. Finally, 1-2-3 Worlds differ greatly from both Dense Worlds and Mazes in that their transitions are drawn from a discrete distribution rather than a continuous one (see Figure A2 in Appendix). Since our work is heavily rooted in the Bayesian approach, the consideration of worlds with a different priors was an important addition to understanding the dependency of an exploration strategy on these priors. 1-2-3 Worlds consist of *N* = 10 states and *M* = 3 actions. In a given state, action *a* = 1 moves the agent deterministically to a single target state, *a* = 2 moves the agent with probability 0.5 to one of two target states, and *a* = 3 moves the agent with probability 0.333 to one of three potential target states. The Appendix contains detailed information on the generative distributions used to create examples from each class of environments and also provides the analytical form for the Bayesian estimate in each world (see Appendix A2).

## 3. Results

### 3.1. Assessing the information-theoretic value of planned actions

The central question to be addressed in this manuscript is how behavior affects the learning process in embodied action-perception loops. The fast reduction of missing information is taken to be the agent's objective during learning-driven exploration (Equation 3). As discussed in section 2.3, the Bayesian estimate minimizes the expected missing information and thus solves the inference problem. The control problem of choosing actions to learn quickly nevertheless remains to be solved. We now show that Bayesian inference can also be used to predict how much missing information will be removed by an action. We call the decrease in missing information between two internal models the *information gain* (I_G_). Letting $\hat{\Theta }$ be a current model derived from data $\vec{\text{d}}$ and ${\hat{\Theta }}^{a,s\to s^{*}}$ be an updated model after observing a transition from *s* to s^*^ under action *a*, the information gain for this observation is:

(6) $$\begin{array}{l} {\text{I}}_{\text{G}}(a,s,s^{*}):={\text{I}}_{\text{M}}(\Theta ∥\hat{\Theta })-{\text{I}}_{\text{M}}(\Theta ∥{\hat{\Theta }}^{a,s\to s}{}^{*})\\ \;=\sum_{s^{\prime }}\Theta_{ass^{\prime }}\log_{2}\frac{{\hat{\Theta }}_{ass^{\prime }}^{a,s\to s*}}{{\hat{\Theta }}_{ass^{\prime }}} \end{array}$$

An exploring agent cannot compute I_G_ directly because it depends on the true CMC kernel Θ. It also cannot know the outcome s^*^ of an action until it has taken it. We therefore again take the Bayesian approach introduced in section 2.3 and consider the agent to treat Θ and s^*^ as random variables. Then, by calculating the expected value of I_G_, we show in Theorem 2 that an agent can compute an estimate of information gain from its prior belief on Θ and the data it has collected. We term this estimate the *predicted information gain* (PIG).

**Theorem 2.** *If an agent is in state s and has previously collected data* $\vec{\text{d}}$*, then the expected information gain for taking action a is given by:*

(7) $$\begin{array}{l} \text{PIG}(a,s):={\text{E}}_{s^{*},\Theta |\vec{\text{d}}}[{\text{I}}_{\text{G}}(a,s,s^{*})]\\ \;=\sum_{s^{*}}{\hat{\Theta }}_{ass^{*}}{\text{D}}_{\text{KL}}({\hat{\Theta }}_{as\cdot }^{a,s\to s^{*}}||{\hat{\Theta }}_{as\cdot }) \end{array}$$

*Proof.* See Appendix A3

PIG has an intuitive interpretation. In a sense the agent imagines the possible outcomes s^*^ of taking action *a* in state *s*. It then determines how each of these results would hypothetically change its internal model ${\hat{\Theta }}^{a,s\to s^{*}}$. It compares these new hypothetical models to its current model by computing the KL-divergence between them. The larger this difference the more information the agent would likely gain if it indeed transitioned to state s^*^. Finally, it averages these hypothetical gains according to the likelihood of observing s^*^ under its current model.

**Figure 2:**
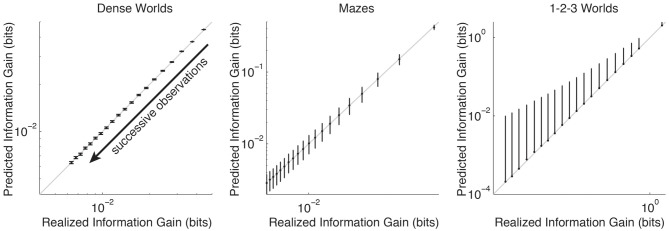
**Accuracy of predicted information gain.** The average predicted information gain is plotted against the average realized information gain. Averages are taken over 200 CMCs, *N* × *M* transition distributions, and 50 trials. Error bars depict standard deviations (only plotted above the mean for 1-2-3 Worlds). The arrow indicates the direction of increasing numbers of observations (top-right = none, bottom-left = 19). The unity lines are drawn in gray.

For each class of environments, Figure 2 compares the average PIG with the average realized information gain as successive observation are used to update a Bayesian estimate. In accordance with Theorem 2, in all three environments PIG accurately predicts the average information gain. Thus, theoretically and empirically, PIG represents an accurate estimate of the improvement an agent can expect in its internal model if it takes a planned action in a particular state.

Interestingly, the expression on the RHS of Equation (7) has been previously studied in the field of Psychology where it was introduced *ad hoc* to describe human behavior during hypothesis testing (Klayman and Ha, 1987; Oaksford and Chater, 1994; Nelson, 2005). To our knowledge, its equality to the predicted gain in information (Theorem 2) is novel. In a later section, we will compare PIG to other measures proposed in the field of Psychology.

### 3.2. Control learners: unembodied and random action

Before introducing and assessing the performance of different explorative strategies, we first develop positive and negative controls. A naive strategy would be to select actions uniformly randomly. Such random policies are often employed to encourage exploration in reinforcement learning models. We will use a *random action* strategy as a negative control exhibiting the baseline learning rate of an undirected explorer.

An *unembodied* agent that achieves an upper bound on expected performance serves as a positive control. Unlike an embodied agent, the unembodied control is allowed, at every time step, to relocate itself to any state it wishes. For such an agent, optimization of learning decomposes into an independent sampling problem (Pfaffelhuber, 1972). Since the PIG for each transition distribution decreases monotonically over successive observations (Figure 2), learning by an unembodied agent can be optimized by always sampling from the state and action pair with the highest PIG. Thus, learning can be optimized in a greedy fashion:

(8) $${\left(a,s\right)}_{\text{Unemb}.}:=\underset{\left(a,s\right)}{\arg\;\max}\text{ PIG}(a,s)$$

**Figure 3:**
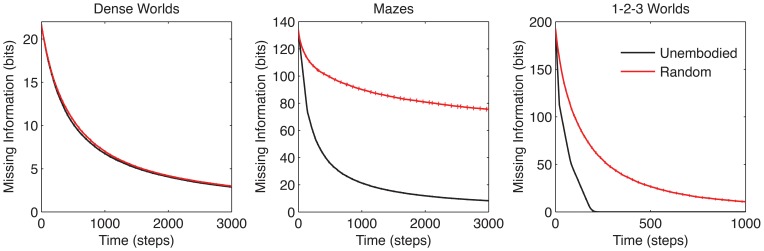
**Learning curves for control strategies.** The average missing information is plotted over exploration time for the unembodied positive control and random action baseline control. Standard errors are plotted as dotted lines above and below learning curves (*n* = 200).

Comparing the learning performances of the random action and unembodied control (red and black curves, respectively in Figure 3) we find a notable difference among the three classes of environments. The performance margin between these two controls is significant in Mazes and 1-2-3 Worlds (*p* < 0.001), but not in Dense Worlds (*p* > 0.01). Despite using a naive strategy, the random actor is essentially reaching maximum performance in Dense Worlds, suggesting that exploration of this environment is fairly easy. In contrast, in Mazes and 1-2-3 Worlds, a directed exploration strategy may be necessary to reach learning speeds closer to the unembodied upper bound.

### 3.3. Exploration strategies based on PIG

PIG represents a utility function that can be used to guide exploration. Since greedy maximization of PIG is optimal for the unembodied agent, one might expect a similar strategy to be promising for an embodied agent. Unlike the unembodied control, however, the embodied agent [PIG(greedy)] would only be able to select its action, not its state:

(9) $$a_{\text{PIG}(\text{greedy})}:=\underset{a}{\arg\;\max}\text{ PIG}(a,s)$$

**Figure 4:**
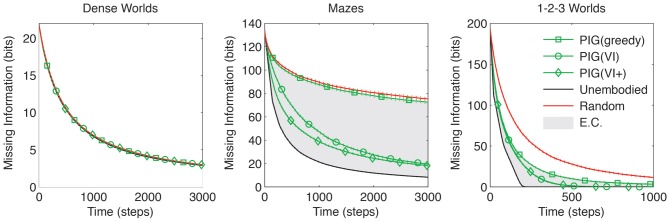
**Coordinating exploration using predicted information gain.** The average missing information is plotted over exploration time for greedy and value-iterated (VI) maximization of PIG. The standard control strategies and the VI+ positive control are also depicted. Standard errors are plotted as dotted lines above and below learning curves (*n* = 200). EC, embodiment constraint.

The performance comparison between PIG(greedy) (Equation 9) and the positive control (Equation 8) is of particular interest because they differ only in that one is embodied while the other is not. As shown in Figure 4 the performance difference is largest in Maze worlds, moderate though significant in 1-2-3 Worlds and smallest in Dense Worlds (*p* < 0.001 for Mazes and 1-2-3 Worlds, *p* > 0.001 for Dense Worlds). To quantify the embodiment constraint faced in a world, we define an *embodiment index* as the relative difference between the areas under the learning curves for PIG(greedy) and the unembodied control. The average embodiment indices for Dense Worlds, Mazes, and 1-2-3 Worlds are 0.02, 2.59, and 1.27, respectively. We also find that, whereas PIG(greedy) yielded no improvement over random action in Dense Worlds and Mazes (*p* > 0.001), it significantly improved learning in 1-2-3 Worlds (*p* < 0.001), suggesting that this utility function was most beneficial in 1-2-3 Worlds.

Greedy maximization of PIG only accounts for the immediately available information gains and fails to account for the effect an action can have on future utility. In particular, when the potential for information gain is concentrated at remote states in the environment, it may be necessary to coordinate actions over time. Forward estimation of total future PIG is intractable. We therefore employ a back-propagation approach previously developed in the field of economics called *value-iteration* (VI) (Bellman, 1957). The estimation starts at a distant time point (initialized as τ = 0) in the future with initial values equal to the PIG for each state-action pair:

$$Q_{0}(a,s):=\text{PIG}(a,s)$$

Then propagating backwards in time, we maintain a running total of estimated future value: 
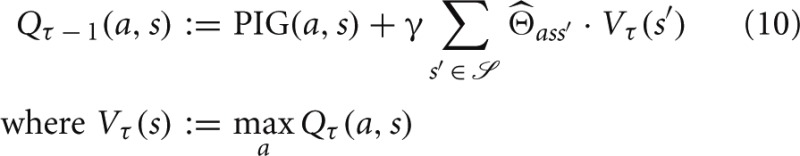


Here, γ is a discount factor, set to 0.95. Such discount factors are commonly employed in value-iteration algorithms to favor more immediate gains over gains further in the future (Bellman, 1957). As discussed later, discounting may also help, in part, to account for the decreasing return on information from successive observations (see Figure 2).

Ideally, the true transition dynamics Θ would be used in Equation (10), but since the agent must learn these dynamics, it employs its internal model $\hat{\Theta }$ instead. Applying the VI algorithm to PIG, we construct a behavioral policy PIG(VI) that coordinates actions over several time steps toward the approximate maximization of expected information gain:

$$a_{\text{PIG}(\text{VI})}:=\underset{a}{\arg\;\max}Q_{-10}(a,s);$$

As shown in Figure 4, the use of VI to coordinate actions yielded the greatest gains in Mazes, with moderate gains also seen in 1-2-3 Worlds. Along with the embodiment indices introduced above, these results support the hypothesis that worlds with high embodiment constraints require agents to coordinate their actions over several time steps to achieve efficient exploration.

Bellman showed that VI accurately estimates future gains when the true transition dynamics Θ is known and when the utility function is stationary (Bellman, 1957). Neither of these are true in our case, and PIG(VI) is therefore only an approximation of future gains. Nevertheless, as we will show, its utility is validated by its superior performance when compared to other previously introduced exploration strategies.

While a learning agent cannot use the true dynamics for VI, we can ascertain how much this impairs its exploration by considering a second positive-control PIG(VI+) which is given the true dynamics for coordinating its actions. That is, this control uses Θ instead of $\hat{\Theta }$ in Equation (10) above. The performance of PIG(VI+) only differs from PIG(VI) in Mazes, and this difference is relatively small compared to the gains made over the random or greedy behaviors (Figure 4). Altogether these results suggest that PIG(VI) may be an effective strategy employable by embodied agents for coordinating explorative actions toward learning.

### 3.4. Structural features of the three worlds

In the course of exploration, the data an agent accumulates will depend on both its behavioral strategy as well as the dynamical structure of its world. To elucidate this interaction, we next consider how structural differences in the three classes of environments correlate with an agents ability to explore. In particular, we consider three structural features of the worlds: their tendency to draw agents into a biased distribution over states, the amount of control a single action provides an agent over its future states, and the average distance between any two states.

#### 3.4.1. State bias

**Figure 5:**
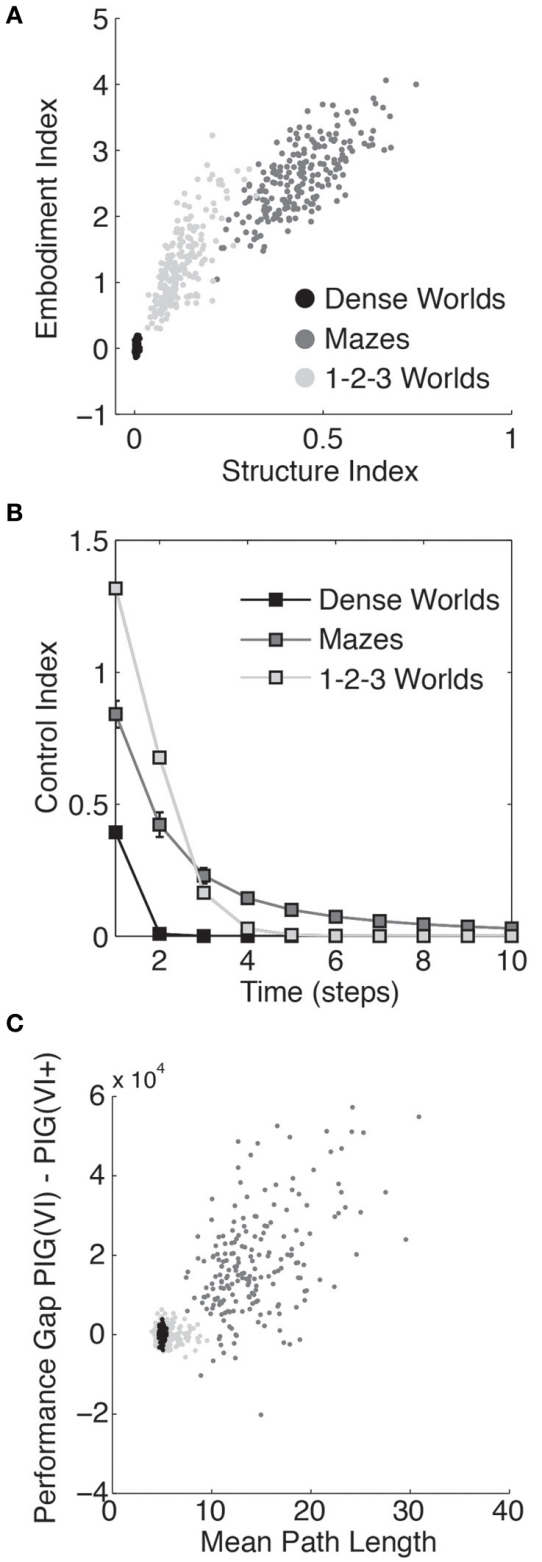
**Quantifying the structure of the worlds. (A)** The embodiment index, defined in section 3.3, is plotted against the structure index for each of 200 Dense Worlds, Mazes, and 1-2-3 Worlds. **(B)** For the same CMCs, the average controllability is plotted as a function of the number of time steps the state lies in the future. The error bars depict standard deviations. **(C)** Again for the same CMCs, the learning performance gap in between PIG(VI) and PIG(VI+) is plotted against the mean path length between any two states.

To assess how strongly a world biases the state distribution of its agents we quantify the unevenness of the equilibrium distribution under a random action policy. The equilibrium distribution Ψ quantifies the likelihood that an agent will be in a particular state at a distant time-point in the future. To quantify the bias of this distribution, we define a *structure index* (SI) as the relative difference between its entropy *H*(Ψ) and the entropy of the uniform distribution *H*(*U*):

$$SI(\Psi ):=\frac{H(U)-H(\Psi )}{H(U)}$$

where: 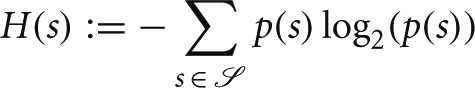


In Figure 5A, the structure indices for 200 worlds in each class of environment are plotted against the embodiment index (defined in section 3.3). As depicted, the embodiment index correlates strongly with the structure index suggesting that state bias represents a significant challenge embodied agents face during exploration.

#### 3.4.2. Controllability

To measure the capacity for an agent to control its state trajectory we computed a control index as the mutual information between a random action *a*_0_ and an agent's state *t* time steps in the future *s*_*t*_ averaged uniformly over possible starting states *s*_0_: 
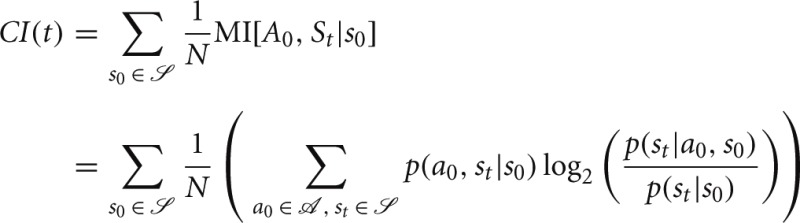


As shown in Figure 5B, an action in a Maze or 1-2-3 Worlds has significantly more impact on future states than an action in Dense Worlds. Controllability is required for effective coordination of actions, such as under PIG(VI). In Mazes, where actions can significantly affect states far into the future, agents yielded the largest gains from coordinated actions. 1-2-3 Worlds also revealed high controllability, but only over the more immediate future. Interestingly, 1-2-3 Worlds also showed moderate gains from coordinating actions.

#### 3.4.3. Mean path length

To assess the size of each CMC, we calculated the average minimum expected path length between every two states. To do this, we first determined the action policy that would minimize the expected path length to any target state. We then calculated the expected number of time-steps it would take an agent to navigate to that target state while employing this optimal policy. The average value of this expected path length taken across start and target states was used as a measure of the extent of the CMC (see Appendix A4 for details). We had previously found that the three classes of CMCs differed in the relative performance between the PIG(VI) explorer and the PIG(VI+) control. Since these two strategies differ only in that the former uses the agent's internal model to coordinate its actions while the latter is allowed to use the true world dynamics, we wondered if the performance gap between the two (the area between their two learning curves) could be related to the path length to a potential source of information. Indeed, comparing this performance gap to the mean path length for each world, we found a strong correlation, as shown in Figure 5C. This suggests that coordination of actions may be more dependent on internal model accuracy for spatially extended worlds. Finally, it is interesting to note that the Mean Path Length is typically larger in mazes than 10 time steps, the planning horizon used in Value Iteration. Ten was chosen simply as a round number and it may be surprising that it works as well as it does in such spatially extended worlds. We believe two factors may contribute to this. First, it is likely that states of high informational value will be close together. Coordinating actions toward a nearby state of high value will therefore likely bring the agent closer to other states of potentially higher value. Second, and we suspect more importantly, since the mean path length is an average, a VI planner can direct its action toward a high information state under the possibility that it might reach that state within 10 time steps even if the expected path length to that location is significantly longer.

### 3.5. Comparison to previous explorative strategies

**Figure 6:**
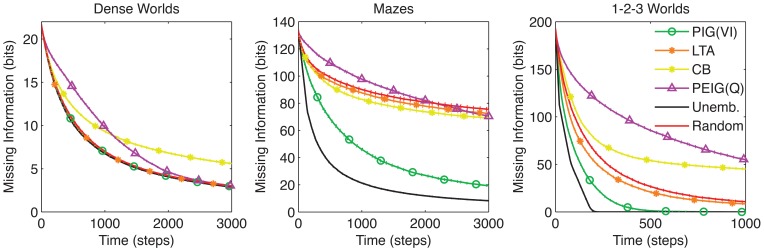
**Comparison to previous exploration strategies.** The average missing information is plotted over time for PIG(VI) agents along with three exploration strategies from the literature: least taken action (LTA) (Sato et al., 1988; Barto and Singh, 1990; Si et al., 2007), counter-based (CB) (Thrun, 1992), and Q-Learning on posterior expected information gain [PEIG(Q)] (Storck et al., 1995). The standard control strategies are also shown. Standard errors are plotted as dotted lines above and below learning curves (*n* = 200).

Models of exploration have been previously developed in the field of reinforcement learning (RL). Usually, these models focus on the role of exploration in reward acquisition rather than its direct role in learning world structure. Still, several of the principles developed in the RL field can be implemented in our framework. In this section, we compare these various methods to PIG(VI) under our learning objective. Since no rewards are available, we consider only RL strategies that can be implemented without rewards. Random action is perhaps the most common exploration strategy in RL. As we have already seen, random action is only efficient for exploring Dense Worlds. The following directed exploration strategies have also been developed in the RL literature (learning curves are plotted in Figure 6):

*Least Taken Action (LTA):* Under LTA, an agent will always choose the action that it has performed least often in the current state (Sato et al., 1988; Barto and Singh, 1990; Si et al., 2007). Like random action, LTA yields uniform sampling of actions in each state. Across worlds, LTA fails to significantly improve on the learning rates seen under random action (*p* > 0.001 for all three environments).
*Counter-Based Exploration (CB):* Whereas LTA actively samples actions uniformly, CB attempts to induce a uniform sampling across states. To do this, it maintains a count of the occurrences of each state, and chooses its action to minimize the expected count of the resultant state (Thrun, 1992). CB performs even worse than random action in Dense Worlds and 1-2-3 Worlds (*p* < 0.001). It does outperform random actions in Mazes but falls far short of the performance seen by PIG(VI) (*p* < 0.001).
*Q-learning on Surprise [PEIG(Q)]:* Storck et al. (1995) developed Surprise as a measure to quantify past changes in an agent's internal model which they used to guide exploration under a Q-learning algorithm (Sutton and Barto, 1998). Interestingly, it can be shown that Surprise as employed by Storck et al. is equivalent to the posterior expected information gain (PEIG), a posterior analog to our PIG utility function (see Appendix A5 and Table 1). Q-learning is a model-free approach to maximizing long-term gains of a utility function (Sutton and Barto, 1998). Implementing this strategy, we found that like CB, PEIG(Q) generally performed worse than random action.

The results in Figure 6 show that PIG(VI) outperforms the previous explorative strategies at learning in structured worlds. We note that all of these strategies were originally developed to encourage exploration for the sake of improving reward acquisition, and their poor performance at our learning objective does not conflict with their previously demonstrated utility under the reinforcement learning framework.

### 3.6. Comparison to utility functions from psychology

Independent findings in Psychology have suggested that the maximization of PIG can be used to predict human behavior during hypothesis testing (Oaksford and Chater, 1994). Inspired by these results, we investigated two other measures also developed in this context. Like PIG, both are measures of the difference between the current and hypothetical future internal models:

*Predicted mode change (PMC)* predicts the height difference between the modes of the current and future internal models (Baron, 2005; Nelson, 2005):
  (11) $$\text{PMC}(a,s)=\sum_{s^{*}}{\hat{\Theta }}_{ass^{*}}\left[\underset{s^{\prime }}{\max}{\hat{\Theta }}_{ass^{\prime }}^{a,s\to s^{*}}-\underset{s^{\prime }}{\max}{\hat{\Theta }}_{ass^{\prime }}\right]$$
*Predicted L1 change (PLC)* predicts the average L1 distance between the current and future internal models (Klayman and Ha, 1987):
  (12) $$\text{PLC}(a,s)=\sum_{s^{*}}{\hat{\Theta }}_{ass^{*}}\left[\frac{1}{N}\sum_{s^{\prime }}\left|{\hat{\Theta }}_{ass^{\prime }}^{a,s\to s^{*}}-{\hat{\Theta }}_{ass^{\prime }}\right|\right]$$

**Figure 7:**
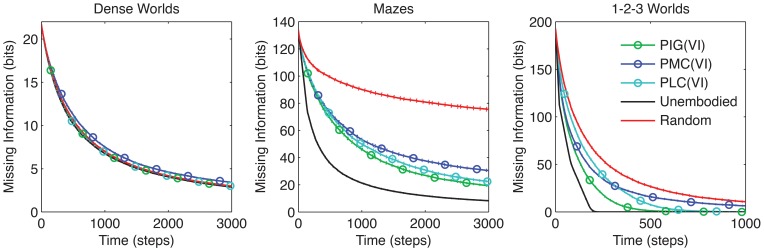
**Comparison between utility functions.** The average missing information is plotted over time for agents that employ VI to maximize long-term gains in the three objective function, PIG, PMC, or PLC. The standard control strategies are also shown (*n* = 200).

We tested agents that approximately maximize PMC or PLC using VI. As Figure 7 reveals, PIG(VI) proved again to be the best performer overall. In particular, PIG(VI) significantly outperforms PMC(VI) in all three environments, and PLC(VI) in 1-2-3 Worlds (*p* < 0.001). Nevertheless, PMC and PLC achieved significant improvements over the baseline control in Mazes and 1-2-3 Worlds, highlighting the benefit of coordinated actions across different utility functions. Interestingly, when performance was measured by an L1 distance instead of missing information, PIG(VI) still outperformed PMC(VI) and PLC(VI) in 1-2-3 Worlds (data not shown).

### 3.7. Generalized utility of exploration

In considering the causes underlying a behavior such as exploration, psychologists often distinguish between the proximate (or behavioral) causes and the ultimate (or evolutionary) causes (Mayr, 1961; Pisula, 2009). Proximate causes are those factors that act directly on the individual in the control of behavior, while ultimate causes are those factors that contribute to the survival value of a behavior upon which natural selection can act. Thus far we have focused on efficient learning as the major objective because it has been identified by psychologists as the primary proximate cause of exploration (Archer and Birke, 1983; Loewenstein, 1994). We now, however, return to the question of the ultimate cause of exploration, which must lie in improved survival or reproductive fitness. The evolutionary advantage of learning-driven exploration is thought to lie in the general usefulness of possessing an accurate internal model of the world (Kaplan and Kaplan, 1983; Renner, 1988, 1990; Pisula, 2003, 2008). Unlike many models of reward-driven exploration, which focus on learning to optimize reward acquisition in a single context, an accurate internal model derived from learning-driven exploration may hold general utility applicable across a wide range of contexts. To compare the general utility of internal models gained through the various exploration methods, we assessed the ability of our agents to apply their internal models toward solving an array of goal-directed tasks. We note that these studies were performed without any changes to the exploration strategies employed by the agent. In essence, we interrupt an agent's exploration at several benchmark time points. We then ask the agent how it would solve, given its internal model, a particular task before allowing it to continue on in its exploration. The agent does not actually perform the task. It is simply asked to solve the task using it internal model. The solution that it provides is then compared by us to the optimal solution. We considered two types of tasks, navigation and reward acquisition:

*Navigation:* Given a starting state, the agent has to quickly navigate to a target state.
*Reward Acquisition:* Given a starting state, the agent has to gather as much reward as possible over 100 time steps. Reward values are drawn from a normal distribution and randomly assigned to every state in the CMC. The agent is given the reward value of each state.

After various lengths of exploration, the agent's internal model is assessed for general utility. For each task, we derive the behavioral policy that optimizes performance under the internal model. As a positive control, we also derive an objective optimal policy that maximizes performance given the true CMC kernel. The difference in realized performance between the agent's policy and the control is used as a measure of navigational or reward loss. For detailed methods, please see Appendix A6.

**Figure 8:**
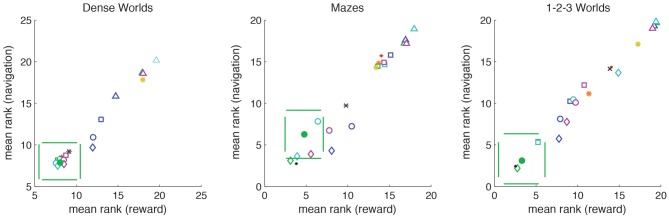
**Demonstration of generalized utility.** For each world (*n* = 200), explorative strategies are ranked for average performance on the navigational tasks (averaged across *N* start states and *N* target states) and the reward tasks (averaged across *N* start states and 10 randomly generated reward distributions). The average ranks are plotted with standard deviations. PIG(VI) is depicted as a filled green circle. Strategies lying outside the pair of horizontal green lines differ significantly from PIG(VI) in navigational performance. Strategies lying outside the pair of vertical green lines differ significantly from PIG(VI) in reward performance (*p* < 0.0001). The different utility functions and heuristics are distinguished by color: PIG(green), PEIG (magenta), PMC (dark-blue), PLC (cyan), LTA (orange), and CB (yellow). The different coordination methods are distinguished by symbol: Greedy (squares), VI (circles), VI+ (diamonds), Heuristic Strategies (asterisks). The two standard controls are depicted as points as follows: Unembodied (black), Random (red). The BOSS reinforcement learner is depicted by a black cross.

Figure 8 depicts the average rank in the navigational and reward tasks for the different explorative strategies. In all environments, for both navigation and reward acquisition, PIG(VI) always grouped with the top performers (*p* > 0.001), excepting positive controls. PIG(VI) was the only strategy to do so. Thus, the explorative strategy that optimized learning under the missing information objective function also prepared the agent for accomplishing arbitrary goal-directed tasks.

Our test for generalized utility differs from the standard reinforcement learning paradigm in that it tests an agent across multiple tasks. The agent therefore cannot simply learn habitual sensorimotor responses specific to a single task. Though most reinforcement learning studies consider only a stationary, unchanging reward structure, we wanted to compare PIG(VI) to reward-driven explorers. BOSS is a state-of-the-art model-based reinforcement learning algorithm (Asmuth et al., 2009). To implement reward-driven exploration we trained a BOSS reinforcement-learner to navigate to internally chosen target-states. After reaching its target, the BOSS agent would randomly select a new target, updating its model reward structure accordingly. We then assessed the internal model formed by a BOSS explorer under the same navigational and reward acquisition tasks. As can be seen in Figure 8, BOSS (black cross) was not as good as PIG(VI) at either class of objectives despite being trained specifically on the navigation task.

## 4. Discussion

In this manuscript we introduced a parsimonious mathematical framework for studying learning-driven exploration by embodied agents based on information theory, Bayesian inference, and CMCs. We compared agents that utilized different exploration strategies toward optimizing learning. To understand how learning performance depends on the structure of the world, three classes of environments were considered that challenge the learning agent in different ways. We found that fast learning could be achieved in all environments by an exploration strategy that coordinated actions toward long-term maximization of PIG.

### 4.1. Caveats

The optimality of the Bayesian estimate (Theorem 1) and the estimation of information gain (Theorem 2) both require an accurate prior over the transition kernels. For biological agents, such priors could have been learned from earlier exploration of related environments, or may represent hardwired beliefs optimized by evolutionary pressures. Alternatively, an agent could attempt to simultaneously learn a prior while exploring its environment. Indeed a simple maximum-likelihood estimation of the concentration parameter for Dense Worlds and Mazes is sufficient for an agent to achieve efficient exploration (data not shown). Nevertheless, biological agents may not always have access to an accurate prior for an environment. For such cases, future work is required to understand exploration under false priors and how they could yield sub-optimal but perhaps biologically realistic exploratory behaviors.

Another potential limitation of our approach occurs from the fact that the VI algorithm is only optimal if the utility function is stationary (i.e., unchanging) (Bellman, 1957). Any utility function, including PIG, that attempts to capture learning progress will necessarily change over time. This caveat may be partially alleviated by the fact that PIG changes only for the sampled distributions. Furthermore, PIG decreases in a monotonic fashion (see Figure 2) which can potentially be captured by the discount factor of VI. Interesting future work may lie in accounting for the effect of such monotonic decreases in estimates of future information gains either through direct estimation or through better approximation by a different choice of discounting mechanism. The problem of accounting for diminishing returns on utility has been previously approached in the field of optimal foraging theory. Modeling the foraging behaviors of animals, optimal foraging theory considers an animals decision of when it should leave its present feeding area, or patch, in which it has been consuming the available food and expend energy to seek out a new, undiminished patch (MacArthur and Pianka, 1966). Charnov's Marginal Value Theorem, a pivotal finding in the field, suggests that the decision to transition should be made once the expected utility of the current patch decreases to the average expected utility across all patches accounting for transition costs (Charnov, 1976). Extending this work to our information-theoretic approach in CMCs may provide the necessary insights to address the challenge of diminishing returns on information gain.

Finally, the VI algorithm scales linearly with the size of the state space, and the calculation of PIG can scale linearly with the square of the size of the state space. This means that for larger and larger CMCs, these approaches will become more computationally expensive to perform. For large worlds, clever methods for approximating these approaches or for sparsifying their representation may be necessary. An explicit model of memory may also be necessary to fully capture the limitation on computational complexity biological organisms face. A wealth of literature from Reinforcement Learning and related fields may offer insights in approaching these challenge which we reserve for future work.

### 4.2. Related work in reinforcement learning

CMCs are closely related to Markov Decision Processes (MDPs) commonly studied in Reinforcement Learning. MDPs differ from CMCs in that they explicitly include a stationary reward function associated with each transition (Sutton and Barto, 1998; Gimbert, 2007). RL research of exploration usually focusses on its role in balancing exploitative behaviors during reward maximization. Several approaches for inducing exploratory behavior in RL agents have been developed. One very common approach is the use of heuristic strategies such as random action, least taken action, and counter-based algorithms. While such strategies may be useful in gathering unchanging external rewards, our results show that they are inefficient for learning the dynamics of structured worlds.

Other RL approaches involve reward-driven exploration. In the absence of external rewards, exploration could still be induced under reward-driven strategies by having the agent work through a series of internally chosen reward problems. This is essentially how the described BOSS agent operates. It was nevertheless insufficient to reach the performance accomplished by PIG(VI).

In addition, several RL studies have investigated intrinsically motivated learning. For example, Singh et al. (2010) have demonstrated that RL guided by saliency, an intrinsic motivation derived from changes in stimulus intensity, can promote the learning of reusable skills. As described in section 3.5, Storck et al. introduced the combination of Q-learning and PEIG as an intrinsic motivator of learning (Storck et al., 1995). In their study, PEIG(Q) outperformed random action only over long time scales. At shorter time scales, random action performed better. Interestingly, we found exactly the same trend, initially slow learning with eventual catching-up, when we applied PEIG(Q) to exploration in our test environments (Figure 6).

### 4.3. Between learning-driven and reward-driven exploration

While curiosity, as an intrinsic value for learning, is believed to be the primary drive of explorative behaviors, other factors, including external rewards, may play a role either in motivating exploration directly or in shaping the development of curiosity (Archer and Birke, 1983; Loewenstein, 1994; Silvia, 2005; Pisula, 2009). In this manuscript, we wished to focus on a pure learning-based exploration strategy and therefore chose to take an unweighted sum of missing information as a parsimonious objective function (Equation 3). Two points, however, should be noted in considering the relationship of this work to previous work in the literature. First, our objective function considers only the learning of the transition dynamics governing a CMC as this fully describes such a world. When we incorporate additional features into our framework, such as rewards in MDPs, those features too could be learned and assessed under our missing information objective function. Toward this goal, interesting insights may come from comparing our work with the multi-armed bandits literature. Multi-armed bandits are a special class of single state MDPs (Gittins, 1979). By considering only a single state, multi-armed bandits remove the embodiment constraint of multi-state CMCs and MDPs. Thus, CMCs and multi-armed bandits represent complimentary special cases of MDPs. That is, a CMC is an MDP without reward structure, while a multi-armed bandit is an MDP without transition kernels. Recent research has attempted to decouple the exploration and exploitation components of optimal control in multi-armed bandits (Abbeel and Ng, 2005; Bubeck et al., 2009). These studies aim at minimizing, through exploration, a construct termed regret, the expected reward forgone by a recommended strategy. Regret is similar to the navigational and reward acquisition loss values we calculated for ranking our explorers under goal-directed tasks. Importantly, while our work considered a wide array of goal-directed tasks, these multi-armed bandit approaches typically consider only learning a single fixed reward structure. Understanding these difference will be important if one wishes to shift attention from the unbiased information-theoretic view we take to a directed task-dependent view. Identifying a means, perhaps through information theory, of quantifying uncertainty in which strategy will optimize a task, will be an important extension bridging these two approaches. The idea of directed information brings us to our second consideration in relating our work to previous literature. Psychologists have found that curiosity, or interest, can vary greatly both between and within individuals (Silvia, 2001, 2006). While one should be careful to not conflate the valuation of an extrinsic reward with the emotion of interest, it is possible such valuations could act to influence the development of interests. By transitioning away from our non-selective measure of missing information toward a weighted objective function that values certain information over others, we may begin to bridge the learning-driven and reward-driven approaches to exploration. One interesting proposal, put forth by Vergassola et al. suggests that information regarding a reward often falls off with distance as an organism moves away from the source of the reward (Vergassola et al., 2007). Accordingly, a greedy local maximization of information regarding the reward may simultaneously bring the individual closer to the desired reward. The resultant “infotaxis” strategy is closely related to our PIG(greedy) strategy but is applied only to a single question of where a particular reward is located.

### 4.4. Related work in psychology

In the Psychology literature, PIG, as well as PMC and PLC, were directly introduced as measures of the expected difference between a current and future belief (Baron, 2005; Klayman and Ha, 1987; Oaksford and Chater, 1994; Nelson, 2005). Here, we showed that PIG equals the expected change in missing information (Theorem 2). Analogous theorems do not hold for PMC or PLC. For example, PLC is not equivalent to the expected change in L1 distance with respect to the true world. This might explain why PIG(VI) outperformed PLC(VI) even under an L1 measure of learning.

We applied PIG, PMC, and PLC to the problem of learning a full model of the world. In contrast, the mentioned psychology studies focussed specifically on hypothesis testing and did not consider sequences of actions or embodied action-perception loops. These studies revealed that human behavior during hypothesis testing can be modeled as maximizing PIG, suggesting that PIG may have biological significance (Oaksford and Chater, 1994; Nelson, 2005). However, those results could not distinguish between the different utility functions (PIG, PMC, and PLC) (Nelson, 2005). Our finding that 1-2-3 Worlds give rise to large differences between the three utility functions may help identify new behavioral tasks for disambiguating the role of these measures in human behavior.

To model bottom–up visual saliency and predict gaze attention, Itti and Baldi recently developed an information-theoretic measure closely related to PEIG (Itti and Baldi, 2006, 2009; Baldi and Itti, 2010). In this model, a Bayesian learner maintains a probabilistic belief structure over the low-level features of a video. Attention is believed to be attracted to locations in the visual scene that exhibit high Surprise. Several potential extensions of this work are suggested by our results. First, it may be useful to model the active nature of data acquisition during visual scene analysis. In Itti and Baldi's model, all features are updated for all location of the visual scene regardless of current gaze location or gaze trajectory. Differences in acuity between the fovea and periphery, however, suggest that gaze location will have a significant effect on which low-level features can be transmitted by the retina (Wässle and Boycott, 1991). Second, our comparison between PIG and PEIG (Figure 6) suggests that predicting future changes may be more efficient than focusing attention only on those locations where change has occurred in the past. A model that anticipates Surprise, as PIG anticipates information gain, may be better able to explain some aspects of human attention. For example, if a moving object disappears behind an obstruction, viewers may anticipate the reemergence of the object and attend that location. Incorporating these insights into new models of visual saliency and attention could be an interesting course of future research.

### 4.5. Information-theoretic models of behavior

Recently information-theoretic concepts have become more popular in computational models of behavior. These approaches can be grouped under three guiding principles. The first principle uses information theory to quantify the complexity of a behavioral policy, with high complexity considered undesirable. Tishby and Polani for example, considered RL maximization of rewards under such complexity constraints (Tishby and Polani, 2011).

The second principle is to maximize a measure called *predictive information* which quantifies the amount of information a known (or past) variable contains regarding an unknown (or future) variable (Tishby et al., 1999; Ay et al., 2008; Still, 2009). Predictive information has also been referred to as *excess entropy* (Crutchfield and Feldman, 2003) and should not be confused with PIG. When the controls of a simulated robot were adjusted such that the predictive information between successive sensory inputs was maximized, Ay et al. found that the robot began to exhibit complex and interesting explorative behaviors (Ay et al., 2008). This objective selects for behaviors that cause the sensory inputs to change often but to remain predictable from previous inputs, and we therefore describe the resulting exploration as stimulation-driven. Such exploration generally benefits from a good internal model but on its own, does not drive fast learning. It is therefore more suitable later in exploration, after a learning-driven strategy, such as PIG(VI), has had a chance to form an accurate model. PIG, in contrast, is most useful in the early stages when the internal model is still deficient. These complimentary properties of predictive information and PIG lead us to hypothesize that a simple additive combination of the two objectives may naturally lead to a smooth transitioning from learning-driven exploration to stimulation-driven exploration, a transition that may indeed be present in human behavior (see section 4.6).

Epsilon machines introduced by Crutchfield and Young (1989) and the information bottleneck approach introduced by Tishby et al. (1999) combine these first two principles of maximizing predictive information and constraining complexity. In particular maximizing the information between a compressed internal variable and the future state progression subject to a constraint on the complexity of generating the internal variable from sensory inputs. Recently, Still extended the information bottleneck method to incorporate actions (Still, 2009).

Finally, the third information-theoretic principle of behavior is to minimize of free-energy, an information-theoretic bound on surprise. Friston put forth this Free-Energy (FE) hypothesis as a unified variational principle for governing both the inference of an internal model and the control of actions (Friston, 2009). Under this principle, agents should act to minimize the number of states they visit. This stands in stark contrast to both learning-driven and stimulation-driven exploration. A learning-driven explorer will seek out novel states where missing information is high, while a stimulation-driven explorer actively seek to maintain high variation in its sensory inputs. Still, reduced state entropy may be valuable in dangerous environments where few states permit survival. The balance between cautionary and exploratory behaviors would be an interesting topic for future research.

### 4.6. Toward a general theory of exploration

With the work of Berlyne (1966), psychologists began to dissect the different motivations that drive exploration. A distinction between play (or diversive exploration) and investigation (or specific exploration) grew out of two competing theories of exploration. As reviewed by Hutt (1970), “curiosity”-theory proposed that exploration is a consummatory response to curiosity-inducing stimuli (Berlyne, 1950; Montgomery, 1953). In contrast, “boredom”-theory held that exploration was an instrumental response for stimulus change (Myers and Miller, 1954; Glanzer, 1958). Hutt suggested that the two theories may be capturing distinct behavioral modes, with “curiosity”-theory underlying investigatory exploration and “boredom”-theory underlying play. In children, exploration often occurs in two stages, inspection to understand what is perceived, followed by play to maintain changing stimulation (Hutt and Bhavnani, 1972). These distinctions nicely correspond to the differences between our approach and the predictive information approach of Ay et al. (2008) and Still (2009). In particular, we hypothesize that our approach corresponds to curiosity-driven investigation, while predictive information a la Ay et al. and Still may correspond with play. Furthermore, the proposed method of additively combining these two principles (section 4.4), may naturally capture the transition between investigation and play seen in children.

For curiosity-driven exploration, there are many varied theories (Loewenstein, 1994). Early theories viewed curiosity as a drive to maintain a specific level of arousal. These were followed by theories interpreting curiosity as a response to intermediate levels of incongruence between expectations and perceptions, and later by theories interpreting curiosity as a motivation to master one's environment. Loewenstein developed an Information Gap Theory and suggested that curiosity is an aversive reaction to missing information (Loewenstein, 1994). More recently, Silvia proposed that curiosity comprises two traits, complexity and comprehensibility (Silvia, 2005). For Silvia complexity is broadly defined, and includes novelty, ambiguity, obscurity, mystery, etc. Comprehensibility appraises whether something can be understood. It is interesting how well these two traits match information-theoretic concepts, complexity being captured by entropy, and comprehensibility by information gain (Pfaffelhuber, 1972). Indeed, PIG might be able to explain the dual aspects of curiosity-driven exploration proposed by Silvia. PIG is bounded by entropy and thus high values require high complexity. At the same time, PIG equals the expected decrease in missing information and thus may be equivalent to expected comprehensibility.

All told, our results add to a bigger picture of exploration in which the theories for its different aspects fit together like pieces of a puzzle. This invites future work for integrating these pieces into a more comprehensive theory of exploration and ultimately of autonomous behavior.

### 4.7. Application toward experimental design

In many ways, scientific research itself epitomizes learning-driven exploration. Like our modeled agents, researchers design experiments to maximize their expected gain in information. Recently, there has been growing interest in automated experimental design. While not every experimental paradigm will fit neatly into our CMC framework, our explorative principles may have direct application to closed-loop neurophysiology. Suppose, for example, we are interested in how ongoing activity within a population of neurons affects their receptive fields. To study this, we would want to measure the neurons' responses to different stimuli and determine how those responses are affected by the activity of the neurons just prior to stimulus presentation. Specific sequences of *priming* stimuli may be necessary to drive the neurons into a particular activation state of ongoing activity in which their responses to a *probe* stimulus could be measured. It may be difficult for a researcher to determine before hand which sequences of stimuli are interesting, but PIG(VI) might offer an automated way of choosing appropriate stimuli on the fly. The ongoing activity of a population of neurons can be treated as the states of the system, and the choice of stimuli as the actions. A closed-loop electrophysiology system controlled by PIG(VI) could investigate not only how the neurons responded to presented stimuli but also how to use the stimuli to prime the neurons into interesting states of ongoing activity for probing.

### Conflict of interest statement

The authors declare that the research was conducted in the absence of any commercial or financial relationships that could be construed as a potential conflict of interest.

## Appendix A

### A1 Proof of Theorem 1

**Claim.** *Consider a CMC random variable* **Θ** *modeling the ground truth environment Θ and drawn from a prior distribution f. Given a history of observations* $\vec{\text{d}}$*, the expected missing information between* **Θ** *and an agent's internal model Φ is minimized by the Bayesian estimate Φ =* $\hat{\Theta }$*. That is:*

$$\hat{\Theta }:={\text{E}}_{\Theta |\vec{\text{d}}}[\Theta ]=\underset{\Phi }{arg\;min}\;{\text{E}}_{\Theta |\vec{\text{d}}}\left[{\text{I}}_{\text{M}}(\Theta ||\Phi )\right]$$

*Proof.* Minimizing missing information is equivalent to independently minimizing the KL-divergence of each transition kernel.

$$\begin{array}{l} \underset{\Phi_{as\cdot }}{\arg\;\min}\;{\text{E}}_{\Theta |\vec{\text{d}}}\left[{\text{D}}_{\text{KL}}\left(\Theta_{as\cdot }||\Phi_{as\cdot }\right)\right]\\ \;=\underset{\Phi_{as\cdot }}{\arg\;\min}\;{\text{E}}_{\Theta |\vec{\text{d}}}\left[\sum_{s^{\prime }}\Theta_{ass^{\prime }}\log_{2}\left(\frac{\Theta_{ass^{\prime }}}{\Phi_{ass^{\prime }}}\right)\right]\\ \;=\underset{\Phi_{as\cdot }}{\arg\;\min}\;{\text{E}}_{\Theta |\vec{\text{d}}}\left[\sum_{s^{\prime }}\Theta_{ass^{\prime }}\log_{2}\Theta_{ass^{\prime }}-\Theta_{ass^{\prime }}\log_{2}\Phi_{ass^{\prime }}\right]\\ \;=\underset{\Phi_{as\cdot }}{\arg\;\min}\;-{\text{E}}_{\Theta |\vec{\text{d}}}\left[\sum_{s^{\prime }}\Theta_{ass^{\prime }}\log_{2}\Phi_{ass^{\prime }}\right]\\ \;=\underset{\Phi_{as\cdot }}{\arg\;\min}\;-\sum_{s^{\prime }}{\text{E}}_{\Theta |\vec{\text{d}}}\left[\Theta_{ass^{\prime }}\right]\log_{2}\Phi_{ass^{\prime }}\\ \;=\underset{\Phi_{as\cdot }}{\arg\;\min}\text{ H}\left[{\text{E}}_{\Theta |\vec{\text{d}}}\left[\Theta_{as\cdot }\right];\Phi_{as\cdot }\right] \end{array}$$

Here H denotes cross-entropy (Cover and Thomas, 1991). Finally, by Gibb's inequality (Cover and Thomas, 1991):

$$\underset{\Phi_{as\cdot }}{\arg\;\min}\text{ H}\left[E_{\Theta |\vec{\text{d}}}\left[\Theta_{as\cdot }\right];\Phi_{as\cdot }\right]={\text{E}}_{\Theta |\vec{\text{d}}}\left[\Theta_{as\cdot }\right]={\hat{\Theta }}_{as\cdot }$$

### A2 Generative distributions and bayesian estimates for the 3 classes of environments

1. *Dense Worlds* correspond to complete directed probability graphs with *N* = 10 states and *M* = 4 actions. An example is depicted in Figure A1. Each transition distribution is independently drawn from a Dirichlet distribution over the standard (*N* − 1)-simplex:
  $$f(\Theta_{as\cdot })=\text{Dir}(\alpha )=\frac{1}{Z(\alpha )}\cdot \prod_{s^{\prime }}\Theta_{ass^{\prime }}{}^{\alpha_{s^{\prime }}-1}$$
  The normalizing constant *Z* brings the area under the distribution to 1:
  $$\begin{array}{l} \;Z(\alpha ):=\int_{\Delta_{N-1}}\prod_{s^{\prime }}\Theta_{ass^{\prime }}{}^{\alpha_{s^{\prime }}-1}d\Theta_{as\cdot }=\frac{\prod_{s^{\prime }}\Gamma (\alpha_{s^{\prime }})}{\Gamma (\sum_{s^{\prime }}\alpha_{s^{\prime }})}\\ \text{where }\Gamma (x):=\int_{0}^{\infty }t^{x-1}e^{-t}\text{d}t \end{array}$$
  The mean of a Dirichlet distribution takes on a simple form:
  $$\int_{\Delta_{N-1}}\Theta_{as\cdot }\frac{\prod_{s^{\prime }}\Theta_{ass^{\prime }}{}^{\alpha_{s^{\prime }}-1}}{Z(\alpha )}\;d\Theta_{as\cdot }=\frac{\alpha }{\sum_{s^{\prime }}\alpha_{s^{\prime }}}$$
  We will assume a symmetric prior setting **α**_s′_ equal to α for all *s*′. The vector form of the Dirichlet distribution will nevertheless still be useful in deriving the Bayesian estimate. The parameter α determines how much probability weight is centered at the midpoint of the simplex and is known as the *concentration factor*. For Dense Worlds, we use a concentration parameter α = 1 which results in a uniform distribution over the simplex.
  To derive an analytic form for the Bayesian estimate of Dense Worlds, we define the matrix ***F*** such that ***F***_ass′_ is a count of the number of times *a*, *s* → *s*′ has occurred in the data. Since each layer ${\hat{\Theta }}_{as}$. of the CMC kernel is independently distributed, its posterior distribution can be computed as follows:
  $$\begin{array}{l} f(\Theta |F)=\frac{\prod_{s^{\prime }}\Theta_{ass^{\prime }}^{F_{ass^{\prime }}}\cdot \prod_{s^{\prime }}\Theta_{ass^{\prime }}{}^{\alpha -1}/Z(\alpha )}{p(F)}\\ \;=\frac{\prod_{s^{\prime }}\Theta_{ass^{\prime }}^{F_{ass^{\prime }}+\alpha -1}}{Z(\alpha )p(F)}=\text{Dir}(F+\alpha ) \end{array}$$
  Thus, the posterior distribution is also Dirichlet and the Bayesian estimate $\hat{\Theta }$ is simply the mean of the distribution:
  (13) $${\hat{\Theta }}_{ass^{\prime }}=\frac{F_{ass^{\prime }}+\alpha }{\sum_{s^{*}}F_{ass^{*}}+\alpha }=\frac{F_{ass^{\prime }}+1}{\sum_{s^{*}}F_{ass^{*}}+1}$$
  In this form, we find that the Bayesian estimate for Dense Worlds is simply the relative frequencies of the observed data with the addition of fictitious counts of size α to each bin. The incorporation of this fictitious observation is referred to as Laplace smoothing and is often performed to avoid over-fitting (Manning et al., 2008). The derivation of Laplace smoothing from Bayesian inference over a Dirichlet prior is a well known result (MacKay and Peto, 1995).
2. *Mazes* consist of *N* = 36 states corresponding to rooms in a randomly generated 6 by 6 maze and *M* = 4 actions corresponding to noisy translations, each biased toward one of the four cardinal directions. An example is depicted in Figure 1. Walking into a wall causes the agent to remain in its current location. Thirty transporters are randomly distributed amongst the walls which lead to a randomly chosen absorbing state (concentric rings in Figure 1). States that are not one step away from the originating state (either directly, through a portal, or against a wall) are assumed to have zero probability of resulting from any action. Transition probabilities for states that are one step away are drawn from a Dirichlet distribution with concentration parameter α = 0.25, and the highest probability is assigned to the state corresponding to the preferred direction of the action. The small concentration parameter distributes more probability weight in the corners of the simplex resulting in less entropic transitions.
  Letting *N*_s_ denote the number of states one-step away from state *s*, the Bayesian estimate for maze transitions is given by:
  (14) $${\hat{\Theta }}_{a,s,s^{\prime }}=\frac{F_{ass^{\prime }}+\alpha }{N_{s}\cdot \alpha +\sum_{s^{*}}F_{ass^{*}}}$$
  As with Dense Worlds, the Bayesian estimate (Equation 14) for mazes is a Laplace smoothed histogram.
3. *1-2-3 Worlds* consists of *N* = 20 states and *M* = 3 actions. In a given state, action *a* = 1 moves the agent deterministically to a single target state, *a* = 2 brings the agent with probability 0.5 to one of two possible target states, and *a* = 3 brings the agent with probability 0.333 to one of three potential target states. The target states are randomly and independently selected for each transition distribution. An absorbing state is form by universally increasing the likelihood that state 1 is chosen as a target. Explicitly, letting Ω_*a*_ be the set of all admissible transition distributions for action *a*:
  $$\Omega_{a}:=\left\{\Theta \in {\mathbb{R}}^{N}|\sum_{s^{\prime }}\Theta_{s^{\prime }}=1\text{ and }\Theta_{s^{\prime }}\in \left\{0,\frac{1}{a}\right\}\forall s^{\prime }\right\}$$
  the transition distributions are drawn from the following distribution:
  (15) $$p(\Theta_{as\cdot })=\{\begin{array}{l} \;\begin{array}{cc} \frac{\begin{array}{c} 0\\ 1-0.75^{a} \end{array}}{\left(\begin{array}{l} N-1\\ a-1 \end{array}\right)} & \begin{array}{c} \text{if }\Theta_{as\cdot }\notin \Omega_{a}\\ \text{ else if }\Theta_{as1}=\frac{1}{a} \end{array} \end{array}\\ \begin{array}{cc} \frac{1-(1-0.75^{a})}{\left(\begin{array}{c} N-1\\ a \end{array}\right)} & \text{otherwise} \end{array} \end{array}$$
  Bayesian inference in 1-2-3 Worlds differs greatly from Mazes and Dense Worlds because of its discrete prior. If *a*, *s* → *s*′ has been previously observed, then the Bayesian estimate for ${\hat{\Theta }}_{ass^{\prime }}$ is given by:
  $${\hat{\Theta }}_{ass^{\prime }}=\frac{1}{a}$$
  If *a*, *s* → *s*′ has not been observed but *a*, *s* → 1 has, then the Bayesian estimate is given by:
  Here *T* is the number of target states that have already been observed. Finally, if neither *a*, *s* → *s*′ nor *a*, *s* → 1 have been observed, then the Bayesian estimate is:
  $${\hat{\Theta }}_{ass^{\prime }}=\{\begin{array}{cc} \frac{1}{a}\cdot \frac{1-0.75^{a}}{1+\left(\left(\begin{array}{c} a-1\\ T \end{array}\right)-1\right)\cdot 0.75^{a}} & \text{if }s^{\prime }=1\\ \frac{1-\left(\frac{T}{a}+{\hat{\Theta }}_{as1}\right)}{N-T-1} & \text{ otherwise} \end{array}$$

### A3 Proof of Theorem 2

**Claim.** *If an agent is in state s and has previously collected data* $\vec{\text{d}}$*, then the expected information gain for taking action a is given by:*

(16) $$\begin{array}{l} \text{PIG}(a,s):={\text{E}}_{s^{*},\Theta |\vec{\text{d}}}[{\text{I}}_{\text{G}}(a,s,s^{*})]\\ \;=\sum_{s^{*}}{\hat{\Theta }}_{ass^{*}}{\text{D}}_{\text{KL}}({\hat{\Theta }}_{as\cdot }^{a,s\to s^{*}}||{\hat{\Theta }}_{as\cdot }) \end{array}$$

*Proof.*

$$\begin{array}{l} {\text{E}}_{s^{*},\Theta |\vec{\text{d}}}[{\text{I}}_{\text{G}}(a,s,s^{*})]={\text{E}}_{s^{*},\Theta |\vec{\text{d}}}\left[\sum_{s^{\prime }}\Theta_{ass^{\prime }}\log_{2}\left(\frac{{\hat{\Theta }}_{ass^{\prime }}^{a,s\to s^{*}}}{{\hat{\Theta }}_{ass^{\prime }}}\right)\right]\\ \;={\text{E}}_{s^{*}|\vec{\text{d}}}\left[\text{​}\sum_{s^{\prime }}{\text{E}}_{\Theta |\vec{\text{d}},s^{*}}\left[\Theta_{ass^{\prime }}\right]\log_{2}\text{​}\left(\frac{{\hat{\Theta }}_{ass^{\prime }}^{a,s\to s^{*}}}{{\hat{\Theta }}_{ass^{\prime }}}\right)\text{​}\right]\\ \;={\text{E}}_{s^{*}|\vec{\text{d}}}\left[\sum_{s^{\prime }}{\hat{\Theta }}_{ass^{\prime }}^{a,s\to s^{*}}\log_{2}\left(\frac{{\hat{\Theta }}_{ass^{\prime }}^{a,s\to s^{*}}}{{\hat{\Theta }}_{ass^{\prime }}}\right)\right]\\ \;={\text{E}}_{s^{*}|\vec{\text{d}}}\left[{\text{D}}_{\text{KL}}({\hat{\Theta }}_{as\cdot }^{a,s\to s^{*}}||{\hat{\Theta }}_{as\cdot })\right]\\ \;=\sum_{s^{*}}p(s^{*}|a,s,\vec{\text{d}}){\text{D}}_{\text{KL}}({\hat{\Theta }}_{as\cdot }^{a,s\to s^{*}}||{\hat{\Theta }}_{as\cdot })\text{ by }(\text{5})\\ \;=\sum_{s^{*}}{\hat{\Theta }}_{ass^{*}}{\text{D}}_{\text{KL}}({\hat{\Theta }}_{as\cdot }^{a,s\to s^{*}}||{\hat{\Theta }}_{as\cdot }) \end{array}$$

### A4 Derivation of mean path length

To optimize navigation to a target state *s*^*^, we consider modified transition probabilities:

$$p_{\text{navigation}}(s^{\prime }|a,s)=\{\begin{array}{ll} \Theta_{ass^{\prime }} & \text{if }s\neq s^{*}\\ \;1 & \text{if }s=s^{\prime }=s^{*}\\ \;0 & \text{otherwise} \end{array}$$

A navigational utility function is then defined as:

$$U_{\text{navigation}}(s)=\{\begin{array}{ll} -1 & \text{if }s\neq s*\\ \;0 & \text{otherwise} \end{array}$$

An optimal policy π is derived through value-iteration as follows:

Value-iteration is continued until *V* converges, and the optimal policy is then defined as:

$$\pi (s)=\underset{a}{\arg\;\max}Q_{\text{convergence}}(a,s)$$

The expected path length to target *s*^*^ is then calculated as:

$$\text{E}[\text{steps to }s^{*}]=\sum_{s}-\frac{1}{N}V_{\text{convergence}}(s)$$

The mean path length is then taken to be the average of the expected path length over the *N* possible target states.

### A5 Derivation of PEIG

**Claim.** *Surprise, as employed by Storck et al. (1995), is equal to the posterior expected information gain. That is, if an agent is in state s and has previously collected data* $\vec{\text{d}}$*, then the expected information gain for taking action a and observing resultant state s^*^ is given by:*

(17) $$\text{Surprise}(a,s,s^{\prime }):={\text{D}}_{\text{KL}}({\hat{\Theta }}_{as\cdot }^{\vec{\text{d}}\cup s^{*}}||{\hat{\Theta }}_{as\cdot }^{\vec{\text{d}}})={\text{E}}_{\Theta |\vec{\text{d}}\cup s^{*}}[{\text{I}}_{\text{G}}(a,s,s^{*})]$$

Proof.

$$\begin{array}{l} {\text{E}}_{\Theta |\vec{\text{d}}\cup s^{*}}[{\text{I}}_{\text{G}}(a,s,s^{*})]={\text{E}}_{\Theta |\vec{\text{d}}\cup s^{*}}\left[\sum_{s^{\prime }}\Theta_{ass^{\prime }}\log_{2}\left(\frac{{\hat{\Theta }}_{ass^{\prime }}^{\vec{\text{d}}\cup s^{*}}}{{\hat{\Theta }}_{ass^{\prime }}^{\vec{\text{d}}}}\right)\right]\\ \;=\sum_{s^{\prime }}{\text{E}}_{\Theta |\vec{\text{d}}\cup s^{*}}\left[\Theta_{ass^{\prime }}\right]\log_{2}\left(\frac{{\hat{\Theta }}_{ass^{\prime }}^{\vec{\text{d}}\cup s^{*}}}{{\hat{\Theta }}_{ass^{\prime }}^{\vec{\text{d}}}}\right)\\ \;=\sum_{s^{\prime }}{\hat{\Theta }}_{ass^{\prime }}^{\vec{\text{d}}\cup s^{*}}\log_{2}\left(\frac{{\hat{\Theta }}_{ass^{\prime }}^{a,s\to s^{*}}}{{\hat{\Theta }}_{ass^{\prime }}^{\vec{\text{d}}}}\right)\\ \;={\text{D}}_{\text{KL}}({\hat{\Theta }}_{as\cdot }^{\vec{\text{d}}\cup s^{*}}||{\hat{\Theta }}_{as\cdot }^{\vec{\text{d}}}) \end{array}$$

### A6 Methods for assessing performance in goal-directed tasks

To assess the general utility of an agent's internal model, the agent is first allowed to explore for a fixed number of times steps. After exploring, the agent is asked, for each goal-directed task, to choose a fixed policy that optimizes performance under its learned model:

*Navigation:* To optimize navigation to a target state *s*^*^ under internal model $\hat{\Theta }$, we took an approach analogous to our method for calculating the mean path length of a world (see Appendix A4). We first consider modified transition probabilities:
  $$p_{\text{navigation}}(s^{\prime }|a,s;\hat{\Theta })=\{\begin{array}{ll} \hat{\Theta }ass^{\prime } & \text{if }s\neq s*\\ \;1 & \text{if }s=s^{\prime }=s*\\ \;0 & \text{otherwise} \end{array}$$
  A navigational utility function is then defined as:
  $$U_{\text{navigation}}(s)=\{\begin{array}{ll} -1 & \text{if }s\neq s*\\ \;0 & \text{otherwise} \end{array}$$
  An optimal policy π_$\hat{\Theta }$_ is derived through value-iteration as follows:
  This process is iterated a number a times, τ_convergence_ > 1000, sufficient to allow *Q* to converge to within a small fixed margin. An optimal policy is then defined as:
  $$\pi_{\hat{\Theta }}(s)=\underset{a}{\arg\;\max}Q_{-\tau_{\text{convergence}}}(a,s)$$
  The realized performance of π_$\hat{\Theta }$_ is assessed as the expected number of time steps, capped at 20, it would take an agent employing π_$\hat{\Theta }$_ to reach the target state. A true optimal policy is calculated as above except using Θ instead of $\hat{\Theta }$. For each world and each exploration strategy, navigation is assessed after *t* ∈ {25, 50, 75, 100, 150, 200, 250, 300, 350, 400, 450, 500, 600, 700, 800, 900, 1000, 1500, 2000, 2500, and 3000} exploration time steps and compared to the true optimal strategy. Performance difference from true optimal is calculated is averaged over the tested exploration lengths, all starting states, and all target states. The different explorative strategies are then ranked in performance.
*Reward Acquisition:* Policies in reward acquisition tasks are derived as above for navigational tasks except as follows:
  $$\begin{array}{l} p_{\text{reward}}(s^{\prime }|a,s;\hat{\Theta })={\hat{\Theta }}_{ass^{\prime }}\\ \;U_{\text{reward}}(s)\sim \text{Uniform}([-1,1])\\ \;\pi_{\hat{\Theta }}(s)=\underset{a}{\arg\;\max}Q_{-100}(a,s) \end{array}$$
  Realized performance is assessed as the expected total rewards accumulated by an agent employing π_$\hat{\Theta }$_ over 100 time steps.
